# Implementing a Practical Global Health Curriculum: The Benefits and Challenges of Patient-Based Learning in the Community

**DOI:** 10.3389/fpubh.2020.00283

**Published:** 2020-07-17

**Authors:** Seema Biswas, Nathan T. Douthit, Keren Mazuz, Zach Morrison, Devin Patchell, Michael Ochion, Leslie Eidelman, Agneta Golan, Michael Alkan, Tzvi Dwolatzky, John Norcini, Igor Waksman, Evgeny Solomonov, A. Mark Clarfield

**Affiliations:** ^1^British Medical Journal Group, BMJ Case Reports, London, United Kingdom; ^2^Brookwood Baptist Health, Medical Education, Birmingham, AL, United States; ^3^British Medical Journal Group, BMJ Case Reports, London, United Kingdom; ^4^Hadassah Academic College, Jerusalem, Israel; ^5^Department of Surgery, Marshfield Medical Center, Marshfield, WI, United States; ^6^Department of Emergency Medicine, Mercy Health, Cincinnati, OH, United States; ^7^Department of Social Work, Municipal Health, Be'er Sheva Municipality, Be'er Sheva, Israel; ^8^Clalit Health Services, Gastroenterology Unit, Ambulatory Specialist Center, Ashkelon, Israel; ^9^Department of Neonatology, Soroka University Medical Center, Be'er Sheva, Israel; ^10^Ben Gurion University of the Negev, Be'er Sheva, Israel; ^11^Department of Medicine, Ben Gurion University of the Negev, Be'er Sheva, Israel; ^12^Medical School for International Health, BGU Faculty for Health Sciences, Be'er Sheva, Israel; ^13^P.H.R. Open Clinic, Volunteer Physician, Tel Aviv-Yafo, Israel; ^14^Geriatric Unit, Rambam Health Care Campus and Faculty of Medicine, Technion - Israel Institute of Technology, Haifa, Israel; ^15^Foundation for Advancement of International Medical Education and Research, Philadelphia, PA, United States; ^16^Psychiatry Department, Upstate Medical University, Syracuse, NY, United States; ^17^Department of Surgery, Galilee Medical Center, Nahariya, Israel; ^18^The Azrieli Faculty of Medicine, Bar Ilan University, Safed, Israel; ^19^Department of General and Hepatobiliary Surgery, Ziv Medical Center, Safed, Israel; ^20^Department of Geriatrics, Soroka University Medical Center, Be'er Sheva, Israel; ^21^Medical School for International Health, Faculty of Health Sciences, Ben-Gurion University of the Negev, Be'er Sheva, Israel; ^22^Department of Geriatrics, McGill University, Montreal, QC, Canada

**Keywords:** global health, medical education in the community, patient-centered curriculum, social determinants of health, global health competencies

## Abstract

**Background:** A growing number of medical schools across the world have incorporated global health (GH) into their curricula. While several schools focus GH education on lecture-based courses, our premise is that global health education should embody a holistic approach to patient care and medical education in local communities. Medical students may learn global health by focusing on real patients, their families and communities as part of a practical curriculum.

**Aims and Objectives:** A unique GH curriculum was devised to compare student learning outcomes on a practical vs. lecture-based course. The premise was that learning from patients would result in a greater breadth of coverage of the global health syllabus as compared to that from a lecture-based course.

**Methods:** A teaching and learning program was developed over 3 years to provide medical students interaction with real patients in the community on a first-preclinical-year Introduction to Global Health and Medical Anthropology course. Learning outcomes on the practical vs. lecture-based course were compared using thematic analysis of the written assignments of both courses: global health case reports and literature reviews, respectively. All members of three cohorts of students undertaking the course in successive academic years were compared (Group A: literature review; Groups B and C: case reports; *n* = 87).

**Results:** Case reports provided evidence of a greater breadth of learning outcomes when compared to the literature review (*p* < 0.001). The writing of the case report was enhanced by completion of a field journal and family health needs assessment tool (*p* < 0.001). Students demonstrated a closeness to their patients that added depth, understanding and motivation to assist patients in health activities and advocate for their needs.

**Discussion:** Placements with patients in the community provided students with a rich learning environment and facilitated the formation of relationships with patients to better understand the social determinants of health and advocate for improvements in their living and working conditions and access to healthcare.

**Conclusions:** Global health may be better learned experientially by following patients rather than from frontal lectures. Patient-based learning inspires a commitment to the individual and facilitates medical schools in meeting their obligations to the communities they serve.

## Introduction

There is growing concern among physicians and medical teachers that medical students have insufficient patient contact and their training is task-focused rather than patient-focused (1–4). Too often they ask “what is the problem?” instead of “how are you?” Following the example of their instructors, they will often see a case or condition instead of a whole person (1, 5–7). In order to make their patients well and keep them well, physicians need to engage effectively with local communities and work to influence all determinants of health: socioeconomic status, how patients live and work; what causes them to become ill; how they live with illness; what influences their recovery; what is required to keep them well; and how to help them exert power over their own health and determinants of health. This is the practice of global health, or put simply, just good medicine; just as clinical medicine is best learned at the bedside (8–12), global health is best learned in the community. Students need to *share* the experiences of their patients in the community (13–16) and medical schools should invest curricular time in the community and honor their social accountability obligations (17–20).

An increasing number of medical schools around the world have incorporated global health into their curricula (21–29). Table 1 lists the learning outcomes expected of the global health curriculum at the Medical School for International Health (MSIH), Ben-Gurion University in Beer Sheva, Israel, where this research was undertaken. MSIH was founded in 1996 as a collaborative effort between Ben-Gurion University and Columbia University Medical Center to produce physicians who, in addition to standard medical training, are required “to acquire competence in [international health and medicine]” (30). Students are mainly from the USA, some are from Canada and a few from outside North America. Teaching is conducted in English. Hebrew language teaching is provided so that students are able to communicate with patients and integrate into the local community.

**Table 1:** MSIH global health core competencies.

**Competency I: Global Health Organizations**	
1. The history of global health	a. Understand the history of global health and the public health interventions that have led to the current state of world health b. Enumerate the major health problems and threats facing the world today and understand how and why they have changed over time c. Understand the difference between public health, global health, and international medicine
2. Global health organization and efforts	a. Understand the history, current role, and efforts of the major global health organizations b. Propose solutions for various problems faced by health workers around the world
3. Health Systems	a. Understand the various structures of healthcare systems b. Understand the application of health services management to lower and middle income countries c. Understand the concept and dimensions of health system performance d. Explore national, inter-organizational, community, and patient level interventions to improve health systems
4. Health economics	a. Understand major financing methods for health care and global health efforts b. Outline and describe key factors in choosing the type of health care financing system c. Understand the major sources of funding in global health and how resources are allotted
5. Health policy	a. Understand how health policies are made and implemented b. Understand how data on global health measures affect policy change and development
6. Politics	a. Understand the importance of local and international politics in the delivery and efficacy of global health and medicine b. Understand different methods and tools that healthcare providers can utilize for political advocacy
**Competency II: Global Burden of Disease**	
1. Determinants of health	a. Understand why it is important to measure health and disease b. Become familiar with various composite measures of burden of disease, their relative strengths and weaknesses, and how they are used in public health literature, World Health Organization reports, and the media c. Apply knowledge of the global burden of disease to the understanding of poverty and global health inequalities
2. Global patterns of morbidity and mortality	a. Understand the attributes of morbidity and mortality as they apply to the burden of disease b. Describe the leading causes of morbidity and mortality around the world in low, middle, and high income countries
3. Epidemiology, biostatistics, and surveillance	a. Understand the measures of morbidity and mortality as they are used globally b. Understand the important contributions of chance, bias, and confounding as potential sources of false epidemiologic associations c. Understand how to interpret tests and how they apply to global health d. Be able to describe the various types of epidemiologic study designs and the measures of association they provide e. Learn to apply field-based epidemiology, including rapid assessment tools
4. Infectious and chronic disease	a. Infectious Disease i) Describe the epidemiology of various infectious diseases and the threat they pose to health around the world ii) Understand the national and international response to emerging and reemerging infectious diseases iii) Understand the history, transmission, diagnosis, prevention, management, and treatment of various infectious diseases at the patient and population level iv) Discuss the current controversies and challenges to controlling diseases in resource-limited settings, and developments expected in the future v) Learn about the impact and prevention of antimicrobial resistance on patients, communities, and global health vi) Know where to find additional resources on tropical and infectious diseases b. Nutrition i) Describe nutrition problems around the world ii) Understand the causes and impact of nutritional problems iii) Identify the signs and symptoms of micronutrient deficiencies and understand approaches to addressing these deficiencies iv) Describe key interventions for malnutrition in various settings v) Identify tools for measuring malnutrition and distinguish between growth monitoring and rapid emergency assessment c. Injury and Global Health i) Understand the global impact of injuries, and their relative importance as a cause of morbidity and mortality worldwide ii) Know the most common categories of intentional and unintentional injuries d. Chronic Disease i) Define chronic disease and understand the world wide current trends ii) Identify the reasons for changes in chronic disease incidence and prevalence iii) Know the range of prevention and treatment strategies for chronic diseases in a range of international settings
5. Environmental health	a. Understand how the geography and climate of a region can impact human health b. Understand the importance of environmental issues such as pollution, natural disasters and climate change and the impact they have on health
**Competency III: Cross-Cultural Medicine**	
1. Cultural sensitivity	a. Understand and appreciate the role that culture plays in the practice of medicine and global health b. Gain skills to better interact with culturally distinct beliefs, attitudes, and practices relating to health and medicine
2. Medical anthropology	a. Understand the practice and theory of medical anthropology and its role in global health and medicine
3. Local diplomacy	a. Gain tools to function within foreign healthcare systems and with different patient populations, governments, and colleagues
4. Language skills	a. Appreciate the importance of language in the practice of medicine and global health b. Work toward a functional level of medically oriented Hebrew for clinical rotations
**Competency IV: Vulnerable Populations**	
1. Maternal and child health	a. Describe the social and economic context of maternal and child health b. Understand the basic terms and definitions of indicators specific to these populations c. Understand the main causes of morbidity and mortality for mothers, neonates, infants, and children e. Distinguish maternal health issues and interventions from other women's health issues f. Identify low-cost, effective, community-based approaches to intervention
2. Disasters, displaced persons, refugees, and terrorism	a. Understand the principles and laws governing international humanitarian assistance b. Understand the basic needs for human survival including water, food, sanitation, and safety c. Know the most common causes of morbidity and mortality in populations affected by conflict, disaster, and terrorism, as well as key assessment strategies and public health interventions d. Understand the roles and limitations of health interventions in conflict mitigation and humanitarian protection e. Be able to apply lessons learned to actual cases involving conflict, disaster, displacement, and terrorism
3. Aging populations	a. Develop an overview of the demography of global aging and its relationship to non-communicable diseases b. Understand how the expanding elderly population will influence global health in the future
4. Mental health	a. Describe the epidemiology and impact of mental health issues on populations b. Understand the economic and social costs of mental illness on populations c. Understand the relationship between mental health and chronic illness d. Recognize the barriers to effective treatment of mental illness
5. Poverty	a. Understand how poverty can affect health and how health problems can result in poverty b. Understand how both absolute and relative poverty act as key determinants of health
**Competency V: Primary Care Medicine**	
1. Primary care in global health	a. Define primary care and understand the way in which it is defined and practiced in different cultures and health systems b. Understand the history of primary care and its global importance today c. Recognize how countries with inadequate primary health care are adversely affected and how stresses are manifested on primary health care providers d. Understand the various elements of a primary health care delivery system, including a referral system and integration with the private sector e. Identify the various ways in which access to primary care can be blocked or facilitated
2. Preventive medicine	a. Understand the role physicians can play in preventing illness in individuals and populations b. Identify strategies and goals of health systems to prevent illness, including education, screening, vaccination, and prophylaxis c. Be familiar with current trends in national and international prevention programs, for infectious and chronic illness
3. Global pediatrics	a. Understand the unique health needs of infants, children and adolescents, and role of the physician in treating these needs b. Be familiar with organizations and programs geared toward child health control and prevention c. Be familiar with global pediatric vaccination recommendations
4. Sexual and reproductive health	a. Understand the unique health conditions and social issues associated with sexual activity and reproduction and the role of the physician in mediating these issues b. Understand the goals and unique challenges of contraceptive use for the prevention of illness and unwanted pregnancy c. Be familiar with global family planning efforts and the impact such initiatives have on populations
5. Access to essential medicines	a. Understand the current definition and suggested list of essential medications as defined by the WHO and Doctors Without Borders b. Be familiar with current efforts and limitations to increase global access to essential medications
**Competency VI: Global Health Ethics**	
1. Research ethics	a. Know the history and evolution of the field of health research ethics (e.g., Nuremberg, Tuskegee, etc.) b. Describe the key tenets of ethical research and know the major ethical issues in research design c. Understand the researcher's responsibility toward research participants and study communities d. Describe issues of informed consent in global health research e. Describe current international guidelines and regulations relating to human subjects research f. Be able to write a research proposal/application for a US IRB and/or local ethics board g. Describe methods for monitoring and compliance in international human subjects research h. Understand current topics and controversies in human subjects research (e.g., genetic research, ownership of personal data including genetic information, and tissue samples, impact of information sharing on eligibility for health insurance, etc.)
2. Clinical decision-making in low resource settings	a. Describe ethical issues in health resource allocation b. Describe ethical dilemmas in low resource settings such as use and re-use of medical supplies (e.g., needles, gloves, etc.) c. Discuss the ethical considerations of triage and treatment prioritization d. Understand how the priorities and moral philosophies of funding agencies may affect clinical work e. Understand ethical considerations of sustainability and resource management
3. Global health equity	a. Describe the meaning of the “right to health” and understand the concept of social justice as it relates to global health b. Describe the difference between equity and equality and how it applies to global health c. Describe major contributing factors to global health disparities
4. Equity in knowledge sharing: open-access and open source resources	a. Understand the ethical reasoning for open source resources b. Understand the concept and rationale for open-source journals c. Understand the technical aspects of open-access technology including mobile data collection, medical records, etc.
5. Advocacy and community consultation/participation	a. Understand the concept of solidarity as it applies to global medical practice b. Describe the ethical implications of local capacity building c. Understand the concept of the physician as activist; understand basic methods employed in global health advocacy d. Know the history of major health advocacy movements (e.g., HIV/AIDS advocacy in the 1970s, 80's, 90's)
6. Human rights	a. Know the basic documents pertaining to global human rights and keep abreast of new developments in the field b. Describe the ethical and human rights issues arising from international trade and the pharmaceutical industry c. Understand the unique problems of health and human rights in post-conflict settings and complex humanitarian emergencies d. Understand the interactions between health and human rights principles and international development strategies e. Understand how to apply health and human rights principles to the other competencies and subcompetencies f. Evaluate health policies and programs within a human rights framework and be able to discuss their human rights implications

**Figure 1:**
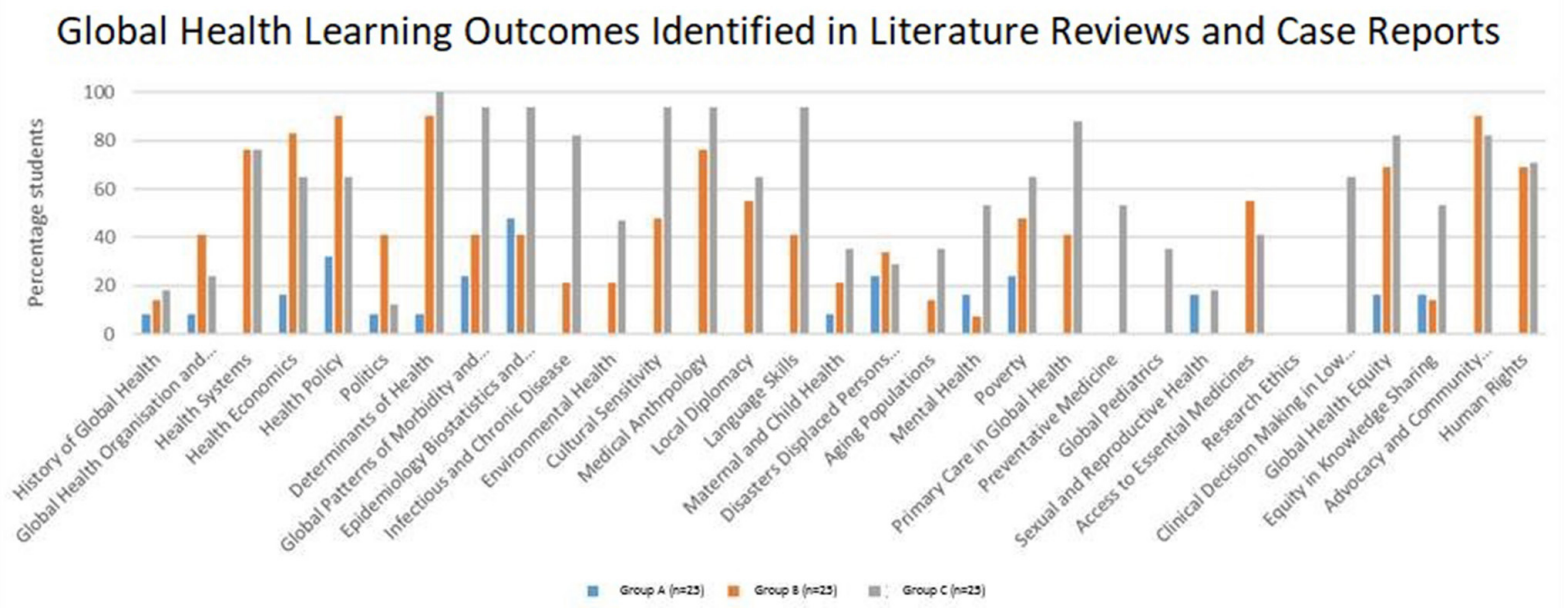
Global health learning outcomes identified in literature reviews and case reports.

The MSIH learning outcomes mirror published Global Health Education Consortium (GHEC) competencies (31) and their achievement is demonstrated in Figure 1. Columns 1 and 2 of Table 2 categorize these competencies in terms knowledge, skills, and attitudes expected of students (32–40). Rather than learning global health primarily through patient contact, most medical school courses are lecture-based, and study global public and population health (23, 25, 26, 37, 41). Students may undertake local or overseas electives in hospitals or community clinics but their personal interaction with patients is usually brief and within the confines of health facilities and clinical environments (42). For students wanting to undertake extended periods of work in communities, global health programs are usually extracurricular. Currently, global health as a concept remains a distinct component of the medical school curriculum rather than a framework integral to all preclinical and clinical medical education (20, 43–45).

**Table 2:** Learning objectives achieved in our practical curriculum.

**Domain**	**Competencies**	**Examples of activities described in field journals or quotations from field journals**
Knowledge and understanding	Knowledge of local history, culture, social structure, politics Understanding the work of local global health practitioners Understanding local healthcare service structure Understanding the impact of migration and marginalization on health	Tour of local community with annotated photographs “We were able to meet with the social worker of Chosen and she gave us some more information about the organization” Investigating local chronic pain services “We talked for about two hours about the circumstances of her life: what her professional life had been like before her retirement, how she felt about her life now”
Skills	Communication and linguistic skills Cross-cultural competence Depth of communication and understanding Assessment of the local burden of disease and health needs of patients, families and communities Sensing vulnerabilities Identification and classification of social and environmental determinants of health Recognition and analysis of health inequalities Problem solving with limited resources Teamwork, strategy and collaborative problem solving Implementation of preventative care Capacity strengthening Advocacy	Learning the patients' language to communicate more effectively with patients “We were nervous, as were the women. For example, one of the women politely asked for permission to smoke” “His mother mentioned that the doctor always asks her before the ultrasound if he has eaten yet and she admitted to me that she always says yes he has. She says that when she feeds him prior to his ultrasound he always vomits when he is laid down on his back” “She depended on the pain clinic for all her pain medication. Although she was not actively receiving care from the clinic beyond obtaining her medications, symbolically the facility was important to her. Whether she was there every day or not, she took comfort in knowing that there existed a health facility in her community that was specifically geared to her and others' pain treatment” “It seemed that this was a very much appreciated and needed break for her and her husband” “We learned about both parents' jobs and how their lives have changed since the patient's birth” “Although this is a medium sized city with all amenities and healthcare clinics available, it is very difficult for someone like her to access them. These are not financial limitations, but rather logistical, on the part of her health plan, which has disheartened our patient to the point where she no longer seems actively willing to seek new forms of care for her pain” Childcare Food and exercise log Monitoring and care of insulin dependent diabetes “We need to figure out how to get a bannister installed in the stairway leading up to the apartment. We must talk to the Ministry of Health to figure this out” Presentations in the classroom and in the community/to relevant global health partners
Attitude	Curiosity Cultural humility Making the most of learning opportunities “in the field” Collaboration and partnership A desire to communicate and get to know the patient Willingness to invest time Engagement in strategies for social justice Ethical reasoning and behavior Professionalism Sincerity and perception Empathy Development of political awareness	“We need to learn more about the Enneagram personality test for our next meeting (one of her interests)” “She was glad that we came to spend some time with her and I think we were able to slightly cheer her up” “Meeting her family gave us a better context about her illness” “I accompanied them to their ultrasound appointment. I met them at their house prior to the appointment and helped them with getting him and his stroller into the car” “We brought two pizzas for the family and our encounter began around some food” “During the visit we worked on his exercises with him…we also did flashcards with him to help with speech development” Racism and difficulties in integration into the community “As a doctor, reassuring the patient of other patients' successes is a huge inspiration of hope (if this is actually true). Otherwise it seems that paying attention to this family is another way that doctors can have a positive influence on the emotional health of the patient's family” “Our goal was to build a stronger relationship with her and her family so that they feel comfortable sharing more intimate information about her health and the impact this has had on her/their lives” “She seems depressed…she half-jokingly said that ninety years is enough. It is possible that she internalises some of the negative emotions of her peers in the establishment” “She admits to having low self-esteem and a negative disposition towards herself due to the lesions and marks caused by the disease. She is worried that she is influencing her daughters in a negative way. She also feels isolated and sometimes ignored” Factors in conflict and migration

We hypothesized that global health is best taught with individual patients central to student learning and propose that patient interaction be fully integrated within the preclinical and clinical biomedical curriculum. We postulated that while students study the presentations and complications of disease, they rarely learn the root causes of disease: the social determinants of health. Thus, at the bedside, students take clinical histories, perform physical examination and follow their patients through their clinical course, and, in the community, students continue the follow up of their patients and see how they cope with ill health and barriers to better health. This teaches students how patients live with disease, respond to treatment and adjust their lifestyles in dealing with the pressures on their health at home, in their local environment, and at work.

In this paper we describe our experience of the implementation of a practical curriculum within an introductory global health course in the first preclinical year in a medical school with a 4-year compulsory global health thread. We attempt to demonstrate the competencies achieved by students on a patient-centered practical curriculum (Table 2) and show that, through focus on an individual patient, the range of global health learning outcomes achieved is far greater than may be achieved on a lecture-based course.

## Materials and Methods

This research was undertaken, with institutional ethics approval, at the Medical School for International Health (MSIH), part of the Ben-Gurion University Faculty of Health Sciences, affiliated with Soroka Hospital in Beer Sheva, Israel. Global health teaching is not fully integrated into all aspects of the 4-year biomedical teaching program at MSIH, but whenever possible is integrated into preclinical and clinical courses, comprising taught courses, specialty modules, workshops, community medicine experience, and a fourth-year international clerkship during which all students complete a 2-month carefully supervised clinical rotation in one of nine sites (in seven low-income countries) with which MSIH has long had close affiliations (46).

This research was conducted over 3 years on the 6-month Introduction to Global Health and Medical Anthropology course spanning the first two semesters of the first preclinical year. Course teaching traditionally comprised lectures on distinct global health topics. The course assignment was a written literature review. The practical course (delivered to 2 consecutive academic years) focused global health learning on student placements with patients in the community. The number of course lectures was reduced and focused on preparing students for their community placements and global health topics arising from student experiences.

### Student Cohorts

Comparison groups for this research comprised the entire class of students on the Introduction to Global Health and Medical Anthropology course in successive academic years. Group A, a class of 24 students studied the hitherto traditional lecture-based course and completed the literature review as their written course assignment. Group B, a class of 29 students, completed the global health case report on the practical course. Group C, a class of 34 students completed the practical course, completed field journals and used the family needs assessment tool (FNAT).

### Developing the Practical Course

**Figure 2:**
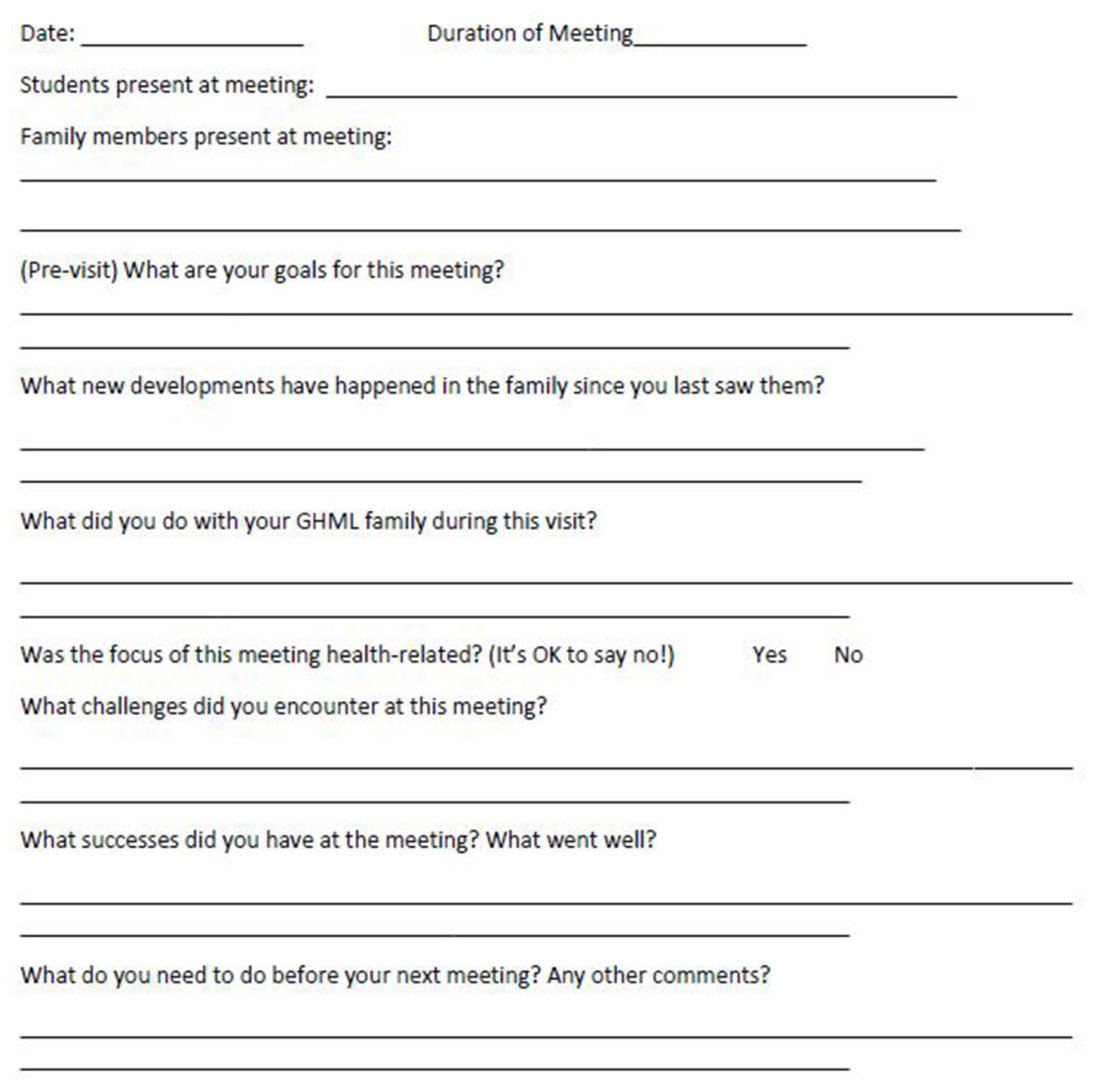
Field journal layout (Group C).

**Figure 3:**
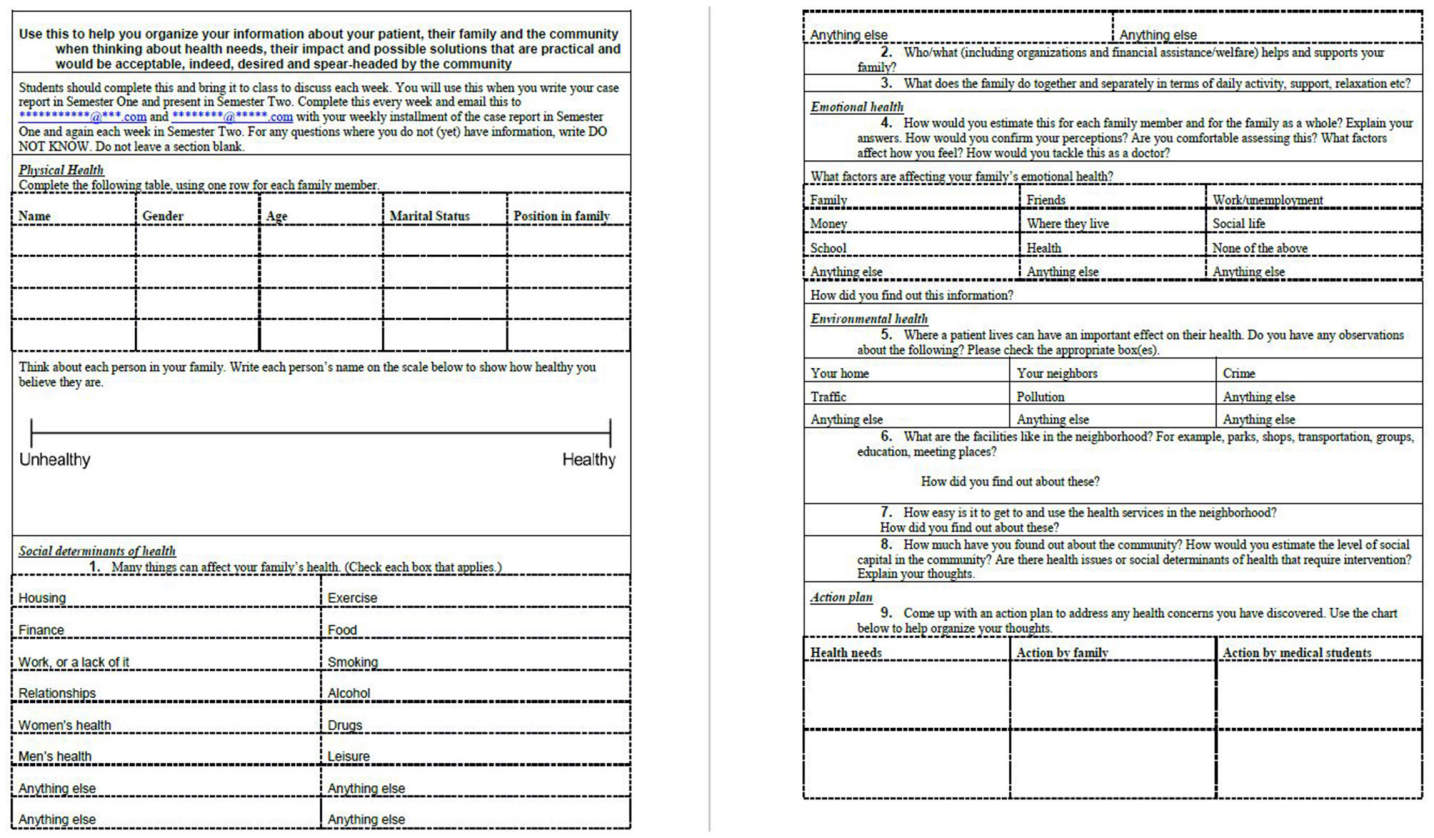
Family Health Needs Assessment Tool [adapted from the WHO family health assessment tool (47)] (Group C).

First-year students undertook 6-month placements with local families or local organizations (Table 3). Placements were identified by a social worker who had worked previously with MSIH students on an extracurricular student placement initiative in the community (Global Health Made Local—GHML), and by Soroka Hospital and community physicians who taught global health at MSIH. Families were selected by the social worker working in partnership with a medical anthropologist faculty member, both of whom had working relationships with local clinicians in the community and in Soroka Hospital. Families with chronic health conditions and documented social care needs were identified and approached months before the course in person by the social worker and/or anthropologist. While selection was not based on ethnicity, the principal of the global health program was to engage with as diverse a selection of local families as possible. This was fairly straightforward as Beer Sheva is home to families of diverse ethnicities and backgrounds and local clinicians facilitated introductions to their patients (families originating from Central Asia and the Indian sub-continent to the Negev desert, and from North America to Africa). Families who consented to involvement in the course formed the basis of a pool of available student placements. Similarly, local community centers, also known to the social worker and medical anthropologist, were approached. MSIH has long had student placements with refugee and migrant health clinics where Soroka clinicians and MSIH faculty practice and supervise students. These clinics were approached and also recruited to the student placement pool. These placements were arranged with the intention to teach students global health outside the classroom and to center learning around individual patients and their families—students following up patients in their home and safe community environments. The social worker and anthropologist carefully outlined the objectives of the program and the level of student interaction expected to families, and shared course outlines and assessment plans with coordinators of the community centers and clinics. This was a dynamic process and concerns about the impact of students on families and the activity of the clinics were incorporated into the guidance and preparation students received, and informed placements of successive student cohorts. Students, working in pairs, were to focus on exploring the cultural, social, environmental, financial and political determinants of health, patients' health beliefs, their approach to living with chronic disease or disability, and access to health care. Placements were with families, community centers, refugee clinics, and assisted living facilities and while selections were not based specifically on gender, age, race, religion, professional or work backgrounds or socioeconomic status, students were matched as closely as possible according to language proficiency and ease of travel. Thus, students proficient in Hebrew were matched with families not proficient in English (as regardless of ethnicity, Hebrew is spoken by all local families including the Bedouin and migrants from India, Africa or the former Soviet Union who participated in the program, for example). GHML students were an important component of the preparatory process for students embarking on placement. Working with the social worker and anthropologist they met with students and mapped out their expectations, language proficiencies, requests to work in pairs (or, rarely, alone) and their concerns regarding travel, expense, and potential time requirements of the course. They worked with students and faculty on the choice of placements and contributed to the support students received during the course. Successive years brought adjustments to the course as we sought to maximize the impact on students, provide benefits to patients and their families and respond to feedback from students, patients, and faculty. In response to calls for more guidance on practical placements, a field journal (Figure 2) and family needs assessment tool (Figure 3) were devised by former GHML students and faculty to help students formulate individual learning objectives, record their findings and set out plans for their next visit. Students (Group C) documented their experiences in field journals including problems they faced on placement.

**Table 3:** Student placements for Groups B and C.

**Local community placement**	**Number of students Group B (total *n* = 29)**	**Number of students Group C (total *n*= 34)**
Families	12	26
Refugee clinic	8	0
Assisted living facilities	0	4
Specialist hospital units	9	0
Community center	0	2
Local NGO	0	2

Formal introductions for the students to their patients were made by course organizers, a team of social workers and GHML volunteers. Families were sent written introductory information about the course and met again with social workers and the clinicians who had recommended them for the program to address outstanding concerns and receive updates on the students they would host. They gave their consent in writing to participate in the program. Formal introductory lectures and GHML volunteer support sessions were set up for the medical students before they met their patients and continued through the first two visits. Students were encouraged to meet their patients within the first 2 weeks of the course and at 2-week intervals thereafter. Assistance was provided with travel expenses as students were mainly reliant on public transport to travel to their placements. All course work, reading material and guidance were available to students online via Moodle, a free and open source online learning management system. Students undertook their field work over 6 months while fulfilling the other obligations of the preclinical biomedical curriculum. Student support over the duration of the course is described in more detail below.

### The Field Journal and Family Health Needs Assessment Tool

The field journal (Figure 2) and family needs assessment tool (FNAT) (Figure 3) were used in the third year of research and put together by former GHML students in collaboration with a social worker and clinical and anthropological faculty. The FNAT was based on the WHO family needs assessment framework (47). Both were designed to provide students with guidance and structure to plan and record their visits to their patients and families, and explore the locality and community services. The aim of both tools was to increase the breadth and depth of knowledge and understanding of global health, assist with faculty feedback and guidance, and inform the case reports that students wrote as their written course assessment tool during the course.

### The Global Health Literature Review

The global health literature review (LR) was a structured essay formerly used as the written assessment tool for the Introduction to Global Health and Medical Anthropology course. The essay involved a comprehensive search and appraisal of the literature in response to a research question formulated by student pairs on the lecture-based global health course.

### The Global Health Case Report

The global health case report (GHCR) was based on the template structure of the clinical case report and structured essay developed for BMJ Case Reports (48, 49). This formed the written assessment tool for the practical (patient-centered) course with a focus on the determinants of health, living with disease and access to health care services.

### Comparing the Lecture-Based and Practical Course

Thematic analysis of case reports and literature reviews was performed to identify learning outcomes from the lecture-based course [assessed through the submission of a literature review—Group A (*n* = 25)] and the patient-based courses [the first class submitting a case report—Group B (*n* = 29)] and the second (subsequent) class submitting a case report after completing field journals and the FNAT—Group C (*n* = 34). The breadth of the syllabus covered through learning outcomes identified on thematic analysis was compared for the case report and literature review.

Thematic analysis was performed by three independent researchers trained in qualitative research method. The analysis of published BMJ global health cases reports (not written by MSIH students) was used to establish inter-rater reliability for learning outcomes in the MSIH global health syllabus (mirroring the GHEC learning outcomes) in Table 1. Learning outcomes were only identified and recorded when these were substantially discussed and referenced in the text. The frequency with which each of these learning outcomes was achieved was quantified and compared for the literature review and case reports (Figure 1). Statistical analysis was performed using Microsoft EXCEL for Mac version 15.7 and SPSS version 23 (SPSS Inc., Chicago, IL, USA). Chi Square Test of significance of frequency was performed to examine the difference between the groups A and B, groups A and C, and groups B and C.

## Results

### Global Health Knowledge, Attitude, and Skills

#### Greater Breadth of GH Knowledge

Based on thematic analysis of literature reviews and case reports, a greater breadth of learning outcomes from the MSIH global health syllabus was achieved on the practical course vs. the lecture-based course (*p* < 0.001 for comparison between groups A and B, and *p* < 0.001 for comparison between groups A and C) (Figure 1).

#### Greater Depth of GH knowledge

**Figure 4:**
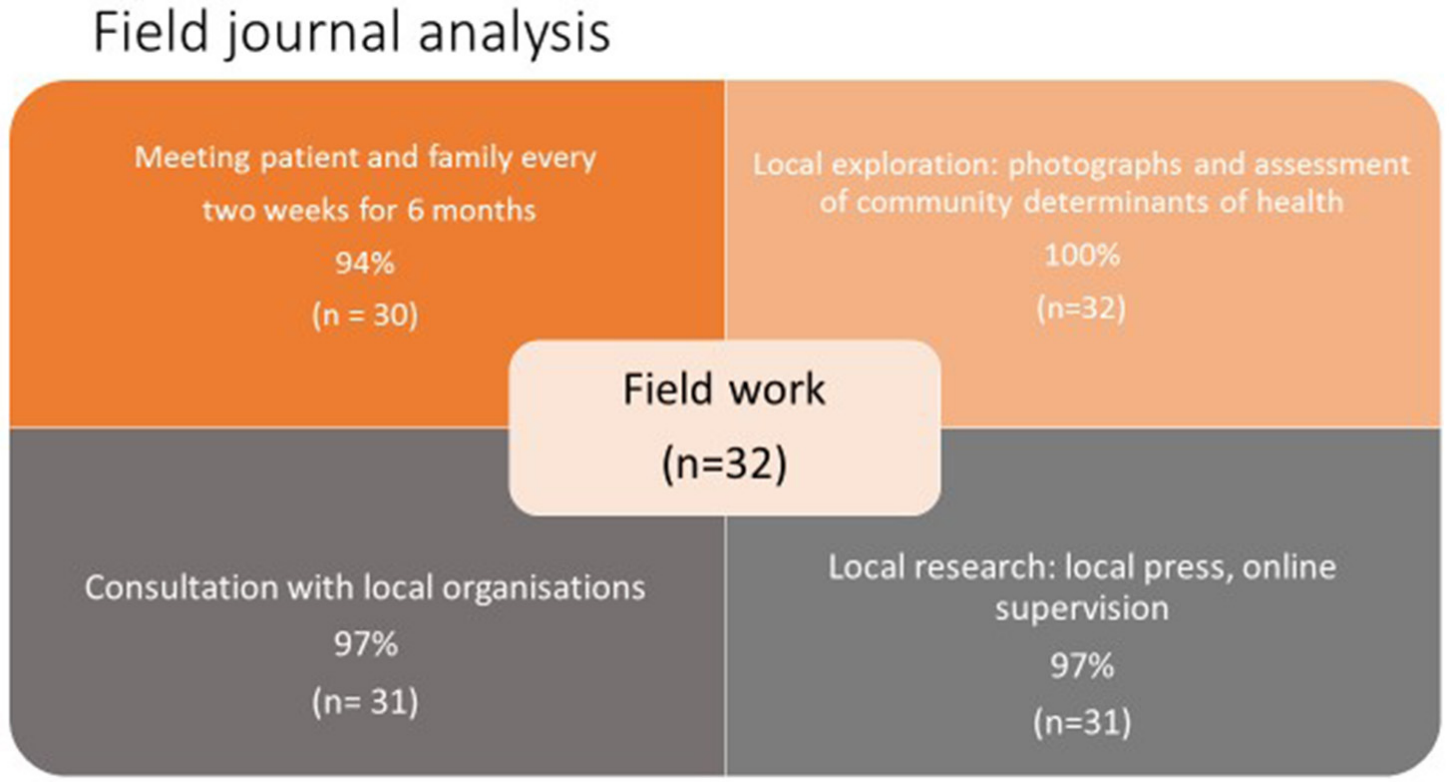
Field journal analysis (Group C).

**Figure 5:**
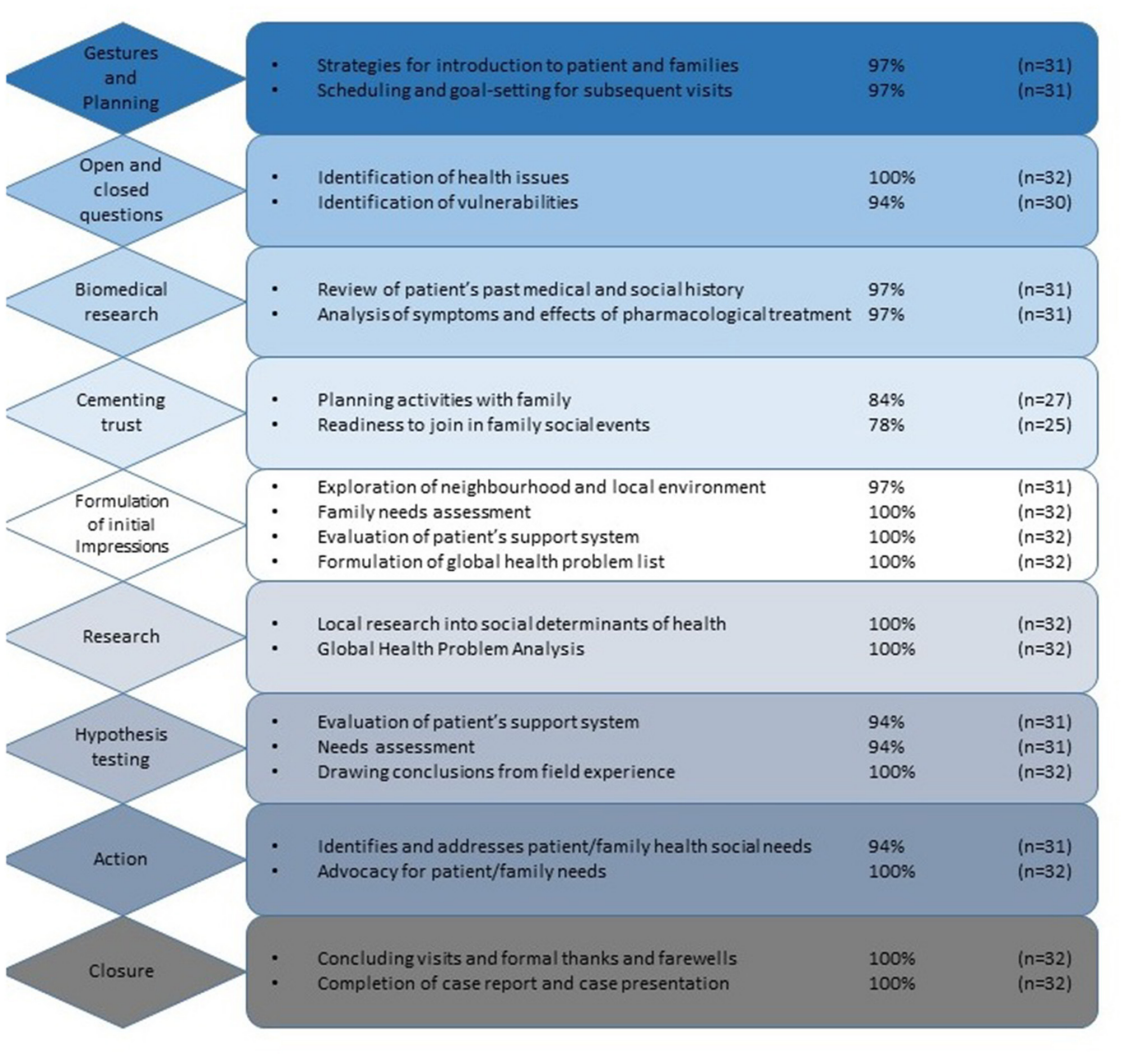
Student strategies in interacting with patients and self-directed learning on placement (Group C).

**Figure 6:**
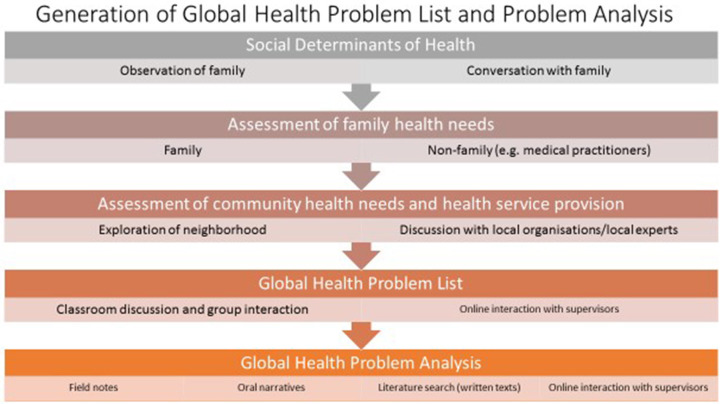
Generation of global health problem list and problem analysis (Group C).

Completing the field journal and FNAT after each visit added depth to each learning outcome (*p* < 0.001 for comparison between groups B and C), i.e., aspects of the syllabus were discussed more frequently and by more students. The contribution of the field journal and FNAT to student learning is further seen in Figures 4, 5. Figure 4 shows how students completed their fieldwork on their practical placements. Much of this was on their own initiative, albeit with guidance from course tutors, and it is likely that, in spite of being allocated curricular time for this, they met their patients, spent “quality” time with them, explored the locality, and researched and consulted local organizations in their own time. Figure 5, put together from analysis of student field journals and the FNAT, shows the process of self-directed student learning in the community. Student numbers and percentages refer to the number of students who adopted a particular strategy or undertook a particular activity and do not refer to how well a task was performed. Students formulated plans and strategized for each visit—looking for answers to questions raised in their field journals and learning more about their patients and their families on each visit. Students undertook activities with their patients and families that brought them closer but also served a health and social need, such as attending doctors' appointments together (Table 2 gives examples from field journal and FNAT entries that illustrate this closeness and the insights that the students gained as a consequence). Information and insights from their field journals and the FNAT informed the global health case reports adding the depth shown in Figure 1. Fieldwork and research were also incorporated into the case report (Figures 4, 6).

Finally, column 3 of Table 2 shows how, through the practical curriculum, students achieved their learning outcomes with examples from field notes, the FNAT and case reports. The knowledge, skills and attitudes of the students were demonstrated on practical placements through each of these reports of their fieldwork.

## Discussion

### Designing and Implementing a Practical Curriculum

We designed a practical program to facilitate student interaction with real patients and in order to deal with the global health problems they encountered in partnership with their patients. The purpose of the practical program was to create an environment for students to learn as much of the global health syllabus as possible, and to learn this in a manner that had meaning for them. The MSIH global health syllabus closely mirrors the GHEC syllabus with learning outcomes widely accepted by medical schools worldwide (31). The premise was that the first-hand study of an individual patient, their family and community would lead students to explore more syllabus topics than might be covered in a didactic lecture-based course. We tested students to discover how much of the syllabus they covered in their learning on the practical vs. lecture-based course by evaluating the learning outcomes achieved. Examples of community learning from community medicine and family medicine programs in the literature were of particular value in developing the course (50).

#### Adding Value for the Students Through a Practical Program

Most medical students begin their medical education with a sincere desire to make a difference in the world (51, 52). They want to help people (14) and they want to learn to become advocates for their patients (3, 14). A practical program offers students the opportunity to focus this enthusiasm on the acquisition of knowledge and the skills necessary to recognize and evaluate global health problems, and to foster an attitude of caring and a closeness to patients that engages students and focuses their efforts on working for their patients. In order to really understand the impact of the social determinants of health to their patients, medical students need to experience these first-hand. Pfeiffer et al. (15), lamenting the production of “education-rich and experience-poor” doctors, describe the lack of first-hand experience for students and doctors in training:

“It's always bothered me that if you want to become a pediatrician, you have to take care of sick children. You can't simply go to class. And yet in schools of public health, we allow people to just go to class, and you don't ever have to take responsibility for a community...We must learn how to get faculty and students responsible for things in the community; that's the way to do hands-on training.”

Thus, as patient interaction remains central to medical education and the clinical curriculum, we argue that patient-based learning should inform as many aspects of the biomedical curriculum as possible (53, 54).

The results of this research show that significantly more of the global health syllabus is learned on the patient-centered practical course (Figure 1), emphasizing the importance of context to learning medicine. Just as we know that students learn clinical medicine better at the bedside (8, 11, 12, 16, 55), patient-based learning in the community is a better way to learn global health. Organization of knowledge acquired in a practical setting is easier (5, 8, 10, 12, 16, 55–58). Students see the relevance of what they learn and have the opportunity to apply what they learn, involve themselves with patients and the community, and take on responsibility, making a constructive contribution toward the care of their patients (11, 59). As Dewachi (60) explains, students “situate their experiences within their socio-political, economic and historical realities” and become sensitized to the realities of public health and standards of living. The field journal and FNAT were designed for students in group C to provide more structure and support on field placements in response to feedback from students in group B. Field journal analysis (Figure 6) illustrates how students consistently developed a context-based learning framework for themselves.

Case reports written with input from the field journal and FNAT (Group C) show that the quality of assessment data is enhanced by a more reflective context (16, 61). Indeed, successive versions of the FNAT tool became more informative as students became more familiar with their patients. Analysis of student field journals (with examples in Table 2) showed that students picked up on both verbal and non-verbal cues and were acutely aware of patient vulnerabilities. Students were moved to put patients at their ease, develop a relationship of trust and helpfulness, while enquiring, researching, and discussing their observations and next steps with course tutors. As students strategized and directed their work, there emerged a clear flow to their thoughts (Figure 6) from the initiation of contact, building relationships, finding answers to their questions, assisting the families where they can, to concluding their interaction at the end of the placement. Course structure and design and student support facilitated this process, but the process was driven by the students and the students sensed how far their interaction should proceed, feeding back their thoughts, and concerns to course tutors in real time. Thus, the field journal and FNAT offered course organizers real time feedback about the interaction of students with patients and their families, and facilitated the resolution of problems with placements early on. Course tutors were able to use the insights of the students to maintain motivation and monitor progress through the course. Students demonstrated empathy, compassion, motivation, advocacy, and leadership in their field journal entries (Table 2—last column and Table 4)—qualities difficult to foster or assess in the classroom. Pfeiffer et al. (15) and Harmer et al. (37) describe the diverse competencies students—“a new generation of multi-talents” (15)—need to acquire through research, training, and service action to become global health leaders of the future. Guiding students through experiential learning was an opportunity to foster in students, not simply the skills to research global health topics, but to assist students in dealing with uncertainty and learn more about the community that would be *their* community while in medical school. The community provides the essential “background” Pfieffer et al. (15) refer to to complement and integrate the biomedical and public health backgrounds required of global health practitioners.

**Table 4:** Quotations from student field journals and the FNAT.

**Building bonds**
“We hope to build foundations for a trusting series of meetings” “We felt a solid basis for future meetings, since she seemed pleased with us and happy that we would come back” “All parties involved began laughing and sharing personal stories while maintaining a sense of purpose related to health issues” “We have good rapport with the family and feel as if they open up to us about their lives” “They also seem to enjoy our visits” “We got to know them on a personal level” “Our goal was to build a stronger relationship with her and her family so that they feel comfortable sharing more intimate information about her health and the impact this has had on her/their lives” “The most crucial aspect is establishing a fruitful relationship with her and her daughter” “We laughed a lot and enjoyed playing cards” “She responded well to the flowers we brought her” “We brought 2 pizzas for the family and our encounter began around some food”
**Learning about local organizations**
“We learned about organisations with which he has been in contact for support with his disease” “We have contacted the event coordinator at the centre to learn more about resources and events to meet more people with CP” “We were able to meet with the social worker of the NGO and she gave us some more information about the organisation”
**Investing time**
“We talked for about two hours about the circumstances of her life: how she came to live here, what her professional life had been like before her retirement, how she felt about her life now” “It may be prudent to do yet more background research and to possibly familiarise ourselves with the neurologist our patient is going to see” “We need to learn more about the Enneagram personality test for our next meeting (one of her interests)” “We need to figure out how to get a bannister installed in the stairway leading up to the apartment. We must talk to the ministry of health to figure this out” “We need to research the riots in Bombay that the family told us about (which seemed to have a profound effect on the patient's psyche)” “She was glad that we came to spend some time with her and I think we were able to slightly cheer her up” “During this visit we worked on his exercises with him…We also did word flashcards with him to help with speech development.” “I accompanied them to their ultrasound appointment. I met them at their house prior to the appointment and helped with getting him and his stroller into the car” “We visited the Bukharic community center” “We played music together...he played with the darbukah” “We learned some French together”
**Living with disease**
“We wanted to ask him more about his limitations due to his disease” “We also learned about the family's challenges with the medical system” “Meeting her family gave us better context about her illness” “We learned about both parents' jobs and how their lives have changed since the patient's birth”
**Picking up on vulnerabilities**
“When speaking of his disease he seems defeated” “Although this is a medium sized city with all the amenities and healthcare clinics available, it is very difficult for someone like her to access them. These are not financial limitations, but rather logistical, on the part of her healthcare plan, which has disheartened our patient to the point where she no longer seems actively willing to seek new forms of care for her pain” “We were nervous as were the women. For example, one of the women politely *asked for permission* to smoke” “Chronic pain is still very much misunderstood making the illness quite physically and psychologically isolating” “She depended on the pain clinic for all her pain medication. Although she was not actively receiving care from the clinic beyond obtaining her medications, symbolically the facility was important to her. Whether she was there every day or not, she took comfort in knowing that there existed a healthcare facility in her community that was specifically geared to hers and others' pain treatment.” “It seemed that this was a very much appreciated and needed break for her and her husband” “The circumstances of her husband's parents made her feel “trapped” “It is a constant worry hanging over them and their family” “She admits to having low self-esteem and a negative disposition towards herself due to the lesions and marks caused by her disease. She is worried that she is influencing her daughters in a negative way. She also feels isolated and sometimes ignored” “She seems depressed…she half-jokingly said that ninety years is enough. It is possible that she internalizes some of the negative emotions of her peers in the establishment”
**Depth**
“We were able to learn more about her family and see how she interacts with them” “If there is one thing that has made her situation bearable, it is her highly supportive group of friends you live in the same community as her and visit her frequently at home” “She mentioned with regards to her pain that her body will reject new chairs, which cause intense pain. Old and familiar furniture in her house, however, cause much less discomfort.” “Older patients who feel unsatisfied with the level of medical attention to their pain management experience poorer health outcomes”
“After having a number of problems and mix-ups involving correspondence with this agency, she worries that one day an approval will not go through and she will be forced to endure potentially severe withdrawal symptoms from not being able to renew her medication on time.” “His mother mentioned that the doctor always asks her before the ultrasound if he has eaten yet and she admitted to me that she always lies and says he has. She says that when she feeds him prior to his ultrasound he always vomits when he is laid down on his back” “As a doctor, reassuring the patient of other patients' successes is a huge inspiration of hope (if this is actually true). Otherwise it seems that paying attention to this family is another way that doctors can have a positive influence on the emotional health of the patient's family” “It is clear that her chronic sickness and the fact that she had to quit her job are difficult for her” “She values control over her situation, and believes that managing her disease at a young age was a crucial step to managing her disease”

#### Adding Value for Patients and the Community

Patients made a substantial commitment in meeting students. They gave of their time, opened their homes to the students and confided in them. Patients gave their personal and familial histories to students as well as their medical histories; they shared their insights and experiences into living with disease and accessing health care. For their part, students attended clinical appointments with patients and were able to advocate for them to some degree within the health system, taking on the role of informal “patient navigators,” facilitating and explaining medical interactions for the patients and offering support and reassurance. A list of the activities students undertook with patients is given in Table 5. As shown in the FNAT, students were encouraged to shop, cook, eat and exercise with the patients and their families, but they undertook activities with the patients and children to enrich the time they spent together and to help the families in their day-to-day activities on their own initiative. Patients welcomed the opportunity to show students how they live and wanted students to see their interactions with health care providers from their point of view. As Sir William Osler wrote, “It is a safe rule to have no teaching without a patient for a text, and the best teaching is taught by the patient himself” (62). Feedback from the patients was strongly positive of the time students spent with them. Feedback from families to course organizers was in the form of confidential informal and formal feedback during and on completion of the placements. Patients, their families, and coordinators at the refugee clinic were unanimous in their opinion that student placements offered important insights for students into how patients cope with health and disease, and that there was much more for students and doctors to understand about their patients outside of the consultation room. Moreover, they felt that the placements were an important bridge, not simply between the students and patients, but the faculty, university, and clinical services as a whole. During the course, however, concerns were raised by families about the length of placements and the frequency of visits, albeit rarely. With successive student cohorts, patient expectations were better managed by faculty. The pool of placements was sufficiently large that only one pair of students be placed with a selected patient over the 2 years.

**Table 5:** Student activities with families (according to field journals).

**Activity with family**	**Percentage of students**
Cooking together	22%
Eating together	88%
Watching TV	22%
Music	46%
Playing cards	22%
Playing board games	46%
Arts and crafts together	22%
Finding out how to help with disease management	100%
Touring town together	88%
Looking through family album	22%
Attending doctors' appointments	68%
Setting up computers	22%
Learning Hebrew	68%
Exercise	68%

Based on the experiences of their patients, students, and faculty alike learned about local and national health care services, medical insurance schemes, and health care coverage, documenting these in their global health case reports (some of which have since been published and contribute to a global evidence base for advocacy). Boelen (63) and Boelen et al. (64) describe medical schools in terms of their social responsibility to the welfare of society; social responsiveness to health priorities in society through education, research, and service; and social accountability in collaboration with community organizations and governments to tackle inequalities in health, the determinants of health, and access to health care. Vulnerable communities that surround academic institutions have been described as the “human capital with whom institutions must partner to achieve educational, clinical and research excellence in health disparities” (13). Practical placements allow medical schools to fulfill their community obligations in terms of social responsibility, responsiveness, and accountability.

### Limitations, Drawbacks, and Challenges

#### Study Limitations

Our study had several limitations. First, the intervention took place in only one institution. Second, the comparison was of three separate cohorts of students, each of which possessed a range of different strengths, weaknesses and qualities amongst individuals. Third, each of these cohorts, though comprising the entire class, was small. While this was an asset in terms of community placement, class discussion and individual support, the small numbers precluded further detailed meaningful statistical analysis. Repeated testing over several cohorts of students or even amongst health and allied health professionals on similar courses would yield more information on the effectiveness of patient-based learning in global health, especially with patient contact for a longer duration than the course permitted. Fourth, it is difficult to assess behavior and attitude in quantitative terms, more so, the evolution of attitudes and transformational learning as students build relationships with patients. We believe that the FNAT and field journal provided useful qualitative information, but these tools will require further development with the input of students and faculty; and indeed, patients. Further, the long-term impact of early patient interaction on students in their clinical years and their careers warrants study. While this was outside the scope of this particular research, plans to follow up former students to gain their perspective on the impact of the practical course in their clinical years and professional lives are underway. Initial feedback is that students had far more insight into the determinants of health and the impact of disease on patients and their families when they subsequently undertook their clinical rotations. Inevitably, this feedback is from former students strongly motivated to pursue global health as practicing doctors who have remained in contact with the authors and continue to collaborate on practical curricula or patient-based learning. A program to follow students through all 4 years of medical school in order to evaluate the impact of the course was not undertaken. Similarly, feedback from clinicians involved in the course who went on to supervise students on their clinical rotations has been sporadic and subjective. Detailed survey of clinical teachers closely observant of student interaction with patients is required to reliably evaluate the impact of the first-year course on the students' clinical years. Finally, we relied on diverse community partnerships across multiple different sites in the community. This introduced variability at odds with the standardization of learning objectives and student experiences; analysis of learning outcomes was, therefore, all the more important.

#### Organizing Placements

A practical course with student placements in the community presents numerous organizational and logistical challenges, but, as shown in this research, pays dividend in the knowledge, skills, and attitudes that students acquire. Most importantly, placements bring students closer to their patients, and their teachers and teaching institutions closer to the community. Placements with patients must be organized thoughtfully, however. It is the university that exerts power in these placements. Patients must be fully informed, be part of the education program and have their needs identified and served as far as possible. Quinn et al. (65) write of the ethical dilemmas of informed consent. Community members must be informed of the risks and benefits of student involvement in their lives and must be able to communicate their needs. One review showed that of 57 articles focused on community-based education, in only 10 articles were community members included in the process of selecting health needs. Further, few articles conceptualized their endeavors as a “collaborative partnership” with the community instead describing their initiatives in terms of “service” or “outreach” (66). Our consent forms were drawn up in collaboration with social workers and produced in two separate local languages (Hebrew and Arabic).

Although the organization of student placements became easier as the practical course became established, ensuring that all students were placed with patients they could travel to easily and communicate with through a shared language was a challenge for course organizers. Ensuring that curricular time was allotted for patient visits was an important factor in planning and motivation for the students who undertook practical work while continuing the courses of the rest of the biomedical curriculum. Providing the structure of the field journal and FNAT substantially improved motivation and self-directed learning, as well as communication with course tutors. Real time student feedback with course tutors was effective in trouble shooting and highlighted student initiative as well as the closeness they had achieved to their patients—students were able to describe situations that were potentially problematic before problems developed and, as a result, adjust accordingly. Examples included, students sensing that patients were beginning to find visits tiring, or family members concerned about the amount of information shared by patients with the students. In each instance, tutors and students were able to mitigate problems: responding to patient feedback about the assistance they really need and planning useful activities together; spacing out visits; or even bringing placements to an end.

Many students in their first year are daunted at the prospect of meeting patients face-to-face, especially in patients' homes. This process must be facilitated by the team of course organizers including social workers, and where possible, an experienced student committee. In this research families were contacted, briefed, and formal introductions made. Almost all students worked in pairs, selecting their partner themselves. Students and families decided together where to meet for the first time, and the timing, venue, and context of their subsequent visits. Patients and students also decided together when their placements would come to a conclusion and a formal closure of the placement was organized with students saying their good byes and the course organizers contacting the patients, checking that their needs were met, listening to feedback, and offering their thanks for participation in the program.

Crossley et al. (67) talks of discovering the worth of a course and improving a course by trial. As the practical course evolved, pitfalls of the course, adjustments in organization, teaching, and the formalization of assessment tools affected both the course that students studied and the research possible. The greatest challenges were in sourcing appropriate placements, settling students in their placements, and managing the expectations of students and local organizations in the first year that the practical curriculum was taught (Group B). Feedback from Group B substantially improved the course for Group C.

#### Administrative Support

The investment in time and planning necessary to ensure the smooth running of the course is substantial. Practical placements require consistent and reliable student support. Course organizers need to ensure that students remain motivated, patients' wishes are accommodated (21, 65) and student supervisors in the community and local organizations remain engaged. The practical course makes considerable demands on faculty and students. The amount of work involved in running a practical curriculum inevitably invites institutional reluctance (68) and concerns over patient and student safety. Benefits of these placements need to be shared with the university administrative team: the teaching of professionalism, understanding the social determinants of health, the impact to local economies, cultural competence, and improvements in public health—all of which affect the community local to the university (66, 69).

Organization of the practical course requires attention to detail and dedication. Course organizers needed to maintain “continuous” contact with the students (students emailed their field work after each visit as instructed but remained in touch with course organizers—mainly via email—every week). Similarly, course organizers remained in touch with social workers and physicians whose patients participated in the program and the patients themselves, troubleshooting where necessary and endeavoring to facilitate the smooth running of the course. There is a good argument for ensuring that course organizers live and work in the same community.

Sourcing and preparation for practical placements involves matching students in terms of language proficiencies and travel distance, preparatory lectures, an explanation of course materials for learning in the community, and the provision of learning resources that students may access easily. The basic requirements of the practical placements are exhaustive, and include: field journals and family health needs assessments; the case report tool; an introduction to library skills; skills in literature search and appraisal (including socio-political, as well as medical literature); clear explanation of the syllabus, course requirements, and learning objectives; assignment deadlines; assigned reading; addressing student concerns; mechanisms for “continuous” real-time feedback; and setting up reliable mechanisms for student support (from faculty and from student peers). In addition, online learning, and support (with all course materials) was set up on the medical school virtual learning platform (Moodle).

Preparatory lectures covered ethical concerns and patient privacy for the students prior to embarking on their placements. They also covered the history and geography of the area in which students would find themselves, useful anthropological concepts to make sense of what they encounter and what to expect on their community visits. Initial contact with patients is not always easy for students. Course organizers and student facilitators need to be able to smooth the introductions and change placements rapidly if students are initially ill-matched or patients are no longer available nor willing to participate in the process.

#### Student Safety

Assurances of student safety during off campus academic activities are a major concern for universities. The steps taken by institutions in preparing their students for international electives are relevant to local placements: pre-placement training should address potential cultural and linguistic barriers; what learners are expected to undertake and what should be avoided; and provide contact information for course organizers and supportive university administrative staff should students need help (70, 71). Institutional legal departments may require the signature of agreements of responsibility and codes of practice or release forms (71, 72). Continuous oversight must be provided by the course organizers in close communication with community supervisors and social work teams to ensure that transport and placements remain safe and effective learning environments (70, 73). Sourcing appropriate placements and establishing reliable mechanisms for continuous feedback and support remain crucial to student safety.

As annual medical school intakes continue to grow, the logistics of sourcing, and overseeing safe and effective practical placements for large numbers of students and assurances for student safety may become prohibitive. The commitment required of course organizers may also become unrealistic. Prohibitive costs of practical programs (37, 74) and educational programs that have successfully substituted learning through interaction with patients with the study of case histories (20) further distance university students from engagement with local populations. The liability of universities for their students' well-being and the substantial administrative commitment mentioned above, tempt medical schools to simulate patient encounters or return to the classroom, rather than work directly with the community (75, 76). There are limits to the educational value of simulation, however, and the benefits to students and the community must ultimately guide the decision to run practical courses (61, 77). More alarming still is the discourse in the literature of social medicine as distinct to the biomedical curriculum (20). We argue for the integration of both. As Kasper et al. (20) assert “all health is global health, all medicine is social medicine.” Placing students outside their traditional comfort zones and encouraging them to deal with uncertainty is also an important part of their education especially in the field of Global Health (8, 11, 12, 16, 55, 56, 78, 79). Students must be provided “with a space in which they feel safe, have control over their actions, enjoy working together, and perceive that their work is making a difference” while teachers need to be able to “generate environments where accepting challenging goals is encouraged” (76).

### Implications for the Wider Biomedical Curriculum

There is a need for more community-based, practical curricula in medical education. Context is crucial to learning medicine—students learn better at the bedside (8, 11, 12, 16, 55). Making sense of their patient encounters is greatly assisted by medical anthropology guidance, and students begin the process of professionalized socialization as they learn, interact and develop their relationships with patients, and adjust and adapt to real-life environments out of the classroom and off campus (79, 80). Experiential learning in the community is transformational as students turn the strange into the familiar and become comfortable in new environments (56, 60, 79). Wass and Mole (16) explain, “medicine is best learned experientially in the clinical environment. Simulation cannot replace it. A doctor works in a stressful uncertain environment where risk and patient safety are of utmost importance. It is virtually impossible to learn in a maximally meaningful way in a non-clinical setting.” There is growing evidence, however, that students spend less time with patients than before (16, 81–85); further, students are rarely observed in their clinical encounters in medical school (86–90). Pre-clinical and basic science courses benefit from early clinical exposure and curricular topics of ethics, communication, and professionalism. Public and global health curricula readily lend themselves to practical learning from real patients, mitigating the limits of simulation, and adding depth to student learning (8, 16, 88). Learning within a practical curriculum “stimulates the students to learn from experience with the aim of facilitating integration of theory and practice” (91). Placements in the community provide the context to understand first-hand the determinants of health and show students the real causes of disease. In the clinical years this becomes even more important as students seek frameworks to understand the effects of disease and strategies in treatment and prevention. Visiting patients at home during clinical rotations potentially adds greatly to student learning and the benefit that patients gain as a result of continuity of care.

Eichbaum's (28) solutions to the deficiencies in global health education are to involve local communities, share learning among individuals and communities, stimulate self-directed learning and opportunities for assessment, and define new competency domains, such as resourcefulness, within the context of local health care delivery. Student placements, shared observations, interactive field journals and the generation and analysis of global health problem lists (as in the global health case report template) would seem a reasonable answer to Eichbaum's analysis of what is needed to improve global health education—especially within local contexts.

Medical schools around the world have a commitment to the production of an informed, impassioned and skilled medical workforce, which has an appreciation of the effects of the determinants of health on communities, is trained in advocacy and humanitarian diplomacy, and has the knowledge and skills needed to enact change locally and globally. Simply put, if clinical medicine is best learnt at the bedside, global health is best learnt in the community. Our research shows that through practical community placements, students are better able to learn global health and its many facets.

## Data Availability Statement

The datasets generated for this study are available on request to the corresponding author.

## Ethics Statement

The studies involving human participants (both in the classroom and the community) were reviewed and approved by Ben Gurion University of the Negev Faculty of Health Sciences affiliated with Soroka Hospital. Written informed consent for participation was not required for this study in accordance with national legislation and institutional requirements.

## Author Contributions

SB designed and researched the material with KM, MA, TD, JN, and AC. MO, ZM, and DP wrote and contributed substantially to GHML materials. AG and LE substantially feedback and informed changes to the practical curriculum. SB and ND wrote the article. All co-authors were involved in revising the article for important intellectual content and gave final approval of the version to be published.

## Conflict of Interest

The authors declare that the research was conducted in the absence of any commercial or financial relationships that could be construed as a potential conflict of interest.
